# Bioemulsification and Microbial Community Reconstruction in Thermally Processed Crude Oil

**DOI:** 10.3390/microorganisms9102054

**Published:** 2021-09-29

**Authors:** Bing Hu, Jie-Yu Zhao, Yong Nie, Xiao-Yu Qin, Kai-Duan Zhang, Jian-Min Xing, Xiao-Lei Wu

**Affiliations:** 1 Group of Biochemical Engineering, Department of Chemical Engineering, College of Chemistry and Chemical Engineering, Beijing Institute of Technology, Beijing 102401, China; binghu319@bit.edu.cn; 2 Key Laboratory of Medical Molecule Science and Pharmaceutics Engineering, Ministry of Industry and Information Technology of China, Beijing 102401, China; 3 College of Engineering, Peking University, Beijing 100871, China; zjyjnzx@163.com (J.-Y.Z.); qinxiaoyu@pku.edu.cn (X.-Y.Q.); zhangkaiduan@pku.edu.cn (K.-D.Z.); 4 CAS Key Laboratory of Green Process and Engineering, Institute of Process Engineering, Chinese Academy of Sciences, Beijing 100190, China; jmxing@ipe.ac.cn; 5 State Key Laboratory of Biochemical Engineering, Institute of Process Engineering, Chinese Academy of Sciences, Beijing 100190, China; 6 Institute of Ecology, Peking University, Beijing 100871, China

**Keywords:** microbial enhanced oil recovery (MEOR), heat perturbation, bioemulsification, microbial community reconstruction, hypersalinity

## Abstract

Utilization of low-cost, environmental-friendly microbial enhanced oil recovery (MEOR) techniques in thermal recovery-processed oil reservoirs is potentially feasible. However, how exogenous microbes facilitate crude oil recovery in this deep biosphere, especially under mesophilic conditions, is scarcely investigated. In this study, a thermal treatment and a thermal recurrence were processed on crude oil collected from Daqing Oilfield, and then a 30-day incubation of the pretreated crude oil at 37 °C was operated with the addition of two locally isolated hydrocarbon-degrading bacteria, *Amycolicicoccus subflavus* DQS3-9A1^T^ and *Dietzia* sp. DQ12-45-1b, respectively. The pH, surface tension, hydrocarbon profiles, culture-dependent cell densities and taxonomies, and whole and active microbial community compositions were determined. It was found that both *A. subflavus* DQS3-9A1^T^ and *Dietzia* sp. DQ12-45-1b successfully induced culture acidification, crude oil bioemulsification, and residual oil sub-fraction alteration, no matter whether the crude oil was thermally pretreated or not. Endogenous bacteria which could proliferate on double heated crude oil were very few. Compared with *A. subflavus*, *Dietzia* sp. was substantially more effective at inducing the proliferation of varied species in one-time heated crude oil. Meanwhile, the effects of *Dietzia* sp. on crude oil bioemulsification and hydrocarbon profile alteration were not significantly influenced by the ploidy increasing of NaCl contents (from 5 g/L to 50 g/L), but the reconstructed bacterial communities became very simple, in which the *Dietzia* genus was predominant. Our study provides useful information to understand MEOR trials on thermally processed oil reservoirs, and proves that this strategy could be operated by using the locally available hydrocarbon-degrading microbes in mesophilic conditions with different salinity degrees.

## 1. Introduction

Since heavy crude oil has significantly higher density and viscosity than light and medium crude oil, it has to be left in hydrocarbon reservoirs until enhanced oil recovery (EOR) techniques are utilized, such as thermal [1], chemical [2], or microbial [3] EOR techniques. Among them, thermal methods are conventional and are most commonly used to enhance oil production [4] because their oil recovery performances are reliable and have no lag phases [1]. In thermal recovery processes, hot water, steam, or gas containing oxygen, which could combust part of the crude oil to release heat, is injected into the oil wells to reduce the viscosity of terrestrial crude oil, to increase the oil volume through thermal expansion, and/or to crack heavy hydrocarbon molecules into smaller ones, allowing crude oil to flow more easily toward production wells [5,6]. However, thermal techniques require a large amount of fresh water and heating energy from fossil fuels, and thus raise environmental and economic concerns [5,7]. Meanwhile, the oil recovery efficiencies of thermal recovery methods are low in mature shale, sandstone, and carbonate reservoirs due to the earlier steam/water breakthrough [8,9]. In recent years, various hybrid methods combining the thermal and chemical EOR processes have been developed to mitigate the negative footprints of thermal oil recovery processes [10,11]. For example, Wu et al. [12] and Liu et al. [13] used foam flooding after hot-water injection in their lab-scale simulated model and sand pack, respectively, and found that this scenario had significantly higher sweep efficiency and oil recovery than hot water flooding individually.

Microbial EOR (MEOR) processes are considered as a potentially low-cost and environment-friendly alternative for other EOR techniques [14]. In in situ MEOR, special microorganisms with or without nutrients are injected into oil reservoirs to facilitate crude oil recovery [15]. During the processes, multiple mechanisms take place at the same time: (1) the introduced microbes might grow exponentially on hydrocarbons and produce byproducts, such as biosurfactants [16,17], biopolymers [18], acids [19], and biogases [20], to alter the physicochemical properties of crude oil in place; (2) the exogenous microbes might degrade the high-viscosity hydrocarbon molecules present in oil reservoirs, such as long-chain alkanes [21], aromatic hydrocarbons [22], resins [17], and asphaltene [23], leading to increased oil sweep efficiencies; and (3) the injected microbial cultures might directly or indirectly stimulate indigenous microorganisms which could produce byproducts or degrade the high-viscosity hydrocarbons [24,25,26,27,28]. Although MEOR processes have a long confinement period before oil production, the MEOR could extract up to 50% of the residual oil in recalcitrant oil reservoirs [29], and thus MEOR field trials have been successfully implemented in different countries for decades [30]. 

Thermal and microbial EOR methods have complementary advantages for oil production, but MEOR techniques being involved in thermal recovery-processed oil reservoirs has seldom been reported. It is inherently considered that heat stress could significantly reduce the total microbial biomass and decrease microbial abundances, especially for fungal communities [31,32], making the reservoirs too sterile to support an effective MEOR process [33]. Nevertheless, because oil layers in oil reservoirs cannot be heated sufficiently during the thermal EOR processes [34], especially in conventional thick heavy oil reservoirs [8], some autochthonous microorganisms were expected to survive for further proliferation, such as methanogenic archaea [35] and thermotolerant bacteria [36]. For example, Rathi et al. [37] enriched an indigenous consortium TERIL146 from a high-temperature recalcitrant oil reservoir in India and found that the consortium flooding had incremental oil recovery after 10 days of incubation in the sand-pack assay. Therefore, it is expected that the integration of thermal and microbial processes is feasible for oil production enhancement. Arora et al. [38] found that 10.1% of residual oil in a hot brine flooding-processed, depleted reservoir model could be recovered through MEOR at 91–96 °C by using a hyperthermophilic bacterial consortium NJS-4 collected from the Ahmadabad and Mehsana oil field in Western India. Nevertheless, it is unknown how thermal treatment and its frequency can negatively affect the microbial community succession in crude oil, especially under the mesophilic conditions. Additionally, there are no reports to show what types of exogenous microbes are suitable to be injected in a thermally process-pretreated oil field for MEOR.

Here, crude oil samples collected from the Daqing Oilfield, China were processed on a thermal treatment and thermal recurrence, followed by the microbial treatment using two bacterial strains, *Amycolicicoccus subflavus* DQS3-9A1^T^ and *Dietzia* sp. DQ12-45-1b. The two strains were isolated from the Daqing Oilfield with different capabilities in hydrocarbon utilization and biosurfactant production. *A. subflavus* DQS3-9A1^T^ was a novel species isolated from oily slurry deposited in the Daqing Oilfield [39], and could grow on alkanes since it contained alkane degrading genes *alk*BGHJKT [40]. *Dietzia* sp. DQ12-45-1b was isolated from the oil production water in Daqing Oilfield [41]. It could effectively utilize n-alkanes (C6–C40), aromatic hydrocarbons, and crude oil to produce biosurfactants by itself [42] or through synergistic interaction with other environmental species [43,44]. Considering that, besides temperature, salinity in the subterranean biosphere is significantly important for microbial growth [45,46] and is various among oil fields in different places [35], whether salinity degree still plays an important role on the microbial community succession and oil physio-chemical characteristic modification after heat perturbation and exogenous microbe injection was investigated in the study. Our results proved that a MEOR trial on thermally pretreated crude oil is feasible if locally available hydrocarbon-degrading and biosurfactant-producing microbes are utilized at mesophilic conditions, regardless of high or low salinity degrees. This study makes up for the deficiencies of MEOR trials in thermally processed oil reservoirs, and provides useful information on characteristics and functions of endogenous and exogenous microorganisms in thermally pretreated crude oil during MEOR.

## 2. Materials and Methods

### 2.1. Oil Sampling, Culture Media and Bacterial Strains

In the study, fresh crude oil samples were taken from an oil well in No.3 oil product of the Daqing Oilfield. The samples were delivered to the laboratory in Beijing through cold-chain transportation in two days, and then were kept at 4 °C for the further processing. 

Here, the bacterial strains utilized as the exogenous microbes were *Amycolicicoccus subflavus* DQS3-9A1^T^, a novel species isolated from oily slurry deposited in the Daqing Oilfield [39], and *Dietzia* sp. DQ12-45-1b, a strong hydrocarbon degrader isolated from the oil production water in the Daqing Oilfield [41]. They were deposited in the China General Microbiological Culture Collection Center (CGMCC, Beijing, China) under accession numbers of 4.3532 and 1.10709, respectively.

Three types of media were used in the study. One was an improved minimal salt medium (I-MSM) with pH 7.0, being used for crude oil microbiome incubation. The composition of I-MSM was as follows: NaCl, 5 g/L or 50 g/L; NH_4_H_2_PO_4_, 1 g/L; (NH_4_)_2_SO_4_, 1 g/L; MgSO_4_·7H_2_O, 0.2 g/L; KNO_3_ 3 g/L; K_2_HPO_4_, 1 g/L; trace element solution (SL-4), 10 mL/L [47]. Another medium was artificial sea water (ASW) with pH 7.0, being used for the preparation of two exogenous bacterial strain seeds. The composition of ASW was: peptone, 5 g/L; yeast extract, 1 g/L; NaCl, 24 g/L; Na_2_SO_4_, 4 g/L; KCl, 0.68 g/L; KBr, 0.1 g/L; H_3_BO_3_, 0.025 g/L; MgCl_2_·H_2_O, 5.4 g/L; CaCl_2_·2H_2_O, 1.5 g/L; SrCl_2_·6H_2_O, 0.024 g/L; NaHCO_3_, 0.2 g/L; Na_2_HPO_4_, 0.04 g/L; NH_4_Cl, 0.5 g/L; NaF, 0.002 g/L [39]. A third medium was Luria-Bertani (LB) solid medium, being used to cultivate and isolate microorganisms in each culture system. The composition of the medium was as follows: peptone, 10 g/L; yeast, 5 g/L; NaCl 10g/L; agar, 18 g/L [39].

### 2.2. Bacterial Seed Preparation

The bacterial strains *A. subflavus* DQS3-9A1^T^ and *Dietzia* sp. DQ12-45-1b were grown in ASW medium at 37 °C for three to five days (OD_600_ ≈ 2.0), respectively. Afterwards, cells were centrifuged and washed with I-MSM medium at 4 °C three times, and then were resuspended in I-MSM to make inoculating seed suspensions.

### 2.3. Oil Heating, Bacterial Inoculation and Incubation

The crude oil floating on production water was centrifuged at 5000 rpm and 4 °C for 20 min to discard the production water. Two grams of the dewatered crude oil samples were added into a 250 mL flask containing 100 mL I-MSM, and then the culture was treated as follows to simulate the thermal process in nature: (1) autoclaving the crude oil culture at 121 °C for 20 min and then cooling down to room temperature; (2) autoclaving at 121 °C for 20 min for two times with a three-day interval, during which the flasks were kept at 37 °C and 200 rpm. To see the effects of the thermal treatments on the microbial communities in crude oil, the flasks containing sterilized I-MSM medium and raw crude oil were taken as the no-thermal pretreatment control. Afterwards, the bacterial seeds of *A. subflavus* DQS3-9A1^T^ and *Dietzia* sp. DQ12-45-1b were separately inoculated into the pretreated crude oil culture with the initial OD_600_ being 0.1. The culture without inoculum was taken as the no-strain control. All cultures were incubated at 37 °C and shaken at a speed of 150 rpm for 30 days. Each treatment was performed in triplicates, and the responding labels are shown in Table 1. During the 30-day cultivation period, 15 mL samples were taken at the 5th, 10th, 20th, and 30th day, each in triplicate, for microbial and chemical data collection.

### 2.4. Physicochemical Property Determination of Culture Solutions and Residual Oil

The culture samples were centrifuged at 8000 rpm and 4 °C for 5 min to separate crude oil and the hydrophilic solutions. For the hydrophilic solutions, they were filtered through 0.22 μm membrane filters (Sartorius Stedim Biotech GmbH, Göttingen, Germany), and then the pH and surface tension of the filtered solutions were determined at room temperature on a pH meter (FH28; Mettler Toledo, Mississauga, ON, Canada) and a Krüss tensiometer (JJ2000B; Shanghai Zhongchen Digital Technic Apparatus Co., Ltd, Shanghai, China), respectively.

A volume of 50 mL of culture samples was taken from each triplicated treatment on Day 0 and Day 30 to determine the hydrocarbon composition profiles. Briefly, 50 mL of solution was added with 0.5 mL HCl solution (6 mol/L), and then the mixture was extracted twice with 20 mL CHCl_3_ in 250 mL separating funnel at 240 rpm for 5 min per time. The lower phase containing CHCl_3_ and extracted oil was transferred into a clean flask, and CHCl_3_ quickly volatized as nitrogen was blown in the fume hood, leaving the residual oil in the flask for the determination of four crude oil subfractions, as described in Gong et al. [47]. The four subfractions were saturated hydrocarbon (SH), aromatic hydrocarbon (AH), non-hydrocarbons (NH), and asphaltenes (ASP). The SH profiles used to indicate the biological degradation of hydrocarbons were determined using a gas chromatography-mass spectrometer (Agilent 5975C-7890A; Agilent Technologies, Santa Clara, CA, USA) according to Tang et al. [48]. In the qualification and quantification process, helium was used as the carrier gas with a flow rate of 1 mL/min, and an HP-5MS column (25 cm × 0.25 mm × 25 µm) was utilized as the separating column. The temperatures of the injector and the detector were both 300 °C. The temperature program was as follows: 40 °C for 10 min, increased by 4 °C/min to 300 °C, and then held at 300 °C for 30 min.

### 2.5. Microbial Isolate Qualification and Quantification in Terms of Colony Forming Unit (CFU)

Culture samples collected on Day 5, Day 10, Day 20, and Day 30 were serially diluted. Then, 0.1 mL of each dilution was transferred on a petri dish containing LB solid medium for cell growth at 37 °C for three to seven days. Microbial colonies in different morphology were counted, isolated, and then incubated in glass tubes containing 3 mL LB liquid medium for pure cultivation at 37 °C and 150 rpm for one to two days. Cells were harvested by centrifugation at 8000 rpm for 5 min, and then the precipitate was processed to obtain genomic DNA by using the GUTC method [42]. The 16S ribosomal RNA (rRNA) genes were amplified with universal bacterial primer pair Eu27F/1492R [48]. Accordingly, the isolates whose 16S rRNA genes had different patterns of terminal-restriction fragment length polymorphism (T-RFLP) digestion could be clustered into different operational taxonomic units (OTUs) [49]. Here, the restriction enzyme *Ras*I (New England BioLabs Inc., Ipswich, MA, USA) was used to digest the amplified 16S rRNA fragments according to Wu et al. [50], and then the digested DNA fragments were separated through electrophoresis on 1% (*w*/*v*) agarose gels (Takara Bio Inc., Dalian, China) to see the T-RFLP digestion patterns for the 16S rRNA gene in each bacterial isolate. The representative isolates were randomly picked and sequenced using high throughput pyrosequencing [51]. The obtained sequences were compared to the SILVA database [52] by local BLASTN algorithm (National Center for Biotechnology Information, Bethesda, MD, USA). The phylogenetic tree was constructed by using the neighbor-joining method on the MEGA7 software [53]. The unit CFU/mL was utilized to calculate cell densities for each isolated OTU. The fourth roots of the relative cell densities in which the absolute cell densities were divided by the least CFU in each OTU were defined, and then the values were set as the diameters of circles indicating the growth trends of each bacterial OTU during the incubation. The total CFU was also calculated to show the total growth-dependent biomass for each time point.

### 2.6. DNA and RNA Extraction

Twenty milliliter culture samples from each treatment were collected at the end the cultivation period, equally divided into two parts, and centrifuged at 8000 rpm and 4 °C for 10 min to collect the precipitates. One part of precipitates was processed with a FastDNA^®^ Spin Kit for Soil (MP Biomedicals, Cleveland, OH, USA) to extract DNA. The DNA of triplicates was mixed for the subsequent microbial community pyrosequencing, the major accurate sequencing platform for long reads [54]. The other part of the precipitates was used for RNA extraction followed by reverse transcription to obtain cDNA fragments according to Nazina et al. [55]. The cDNA suspensions of each triplicate was also mixed and prepared for microbial community pyrosequencing.

### 2.7. Sequencing Library of Bacterial V3–V6 Variable Region of the 16S Ribosomal RNA Gene

For bacterial community analysis, the ~733 bp bacterial V3–V6 variable region of the 16S rRNA gene was amplified from the DNA and cDNA samples with the forward primer 341F+RL** containing the ‘A’ sequences of 454 Life Sciences adaptor and a unique 10 bp optional multiplex identifier (MID) tag (5′-CCATCTCATCCCTGCGTGTCTCCGACTCAGNNNNNNNNNNTCCTACGGGAGGCAGCAG-3′) and the reverse primer 1073R containing the ‘B’ sequences of 454 Life Sciences adaptor (5′-CCTATCCCCTGTGTGCCTTGGCAGTCTCAGACGAGCTGACGACARCCA TG-3′). A duplicate of 50 μL polymerase chain reaction (PCR) mixture contained 10 μL of 5× Pfu Buffer (Beijing Dingguochangsheng Biotech Co., Ltd, Beijing, China), 5 μL of dNTP (2.5 mmol/L; Takara Bio Inc., Dalian, China), 1.25 μL of each primer (5 mmol/L; Invitrogen, Shanghai, China), 1 μL of TransStartTM FastPfu DNA polymerase (2.5 units/μL; TransGen Biotech, Beijing, China) and 30–50 ng of DNA or cDNA templates. PCR process was performed in a PTC-200 thermocycler (Bio-Rad, Munich, Germany) with the following program: 95 °C for 2 min, followed by 20 cycles of denaturation at 95 °C for 30 s, annealing at 56 °C for 30 s, and extension at 72 °C for 30 s; finally, extension at 72 °C for 5 min and preserving at 4 °C. Negative controls without template were always performed to check the performance without contamination. The amplicons of each sample were mixed and re-collected through 1% (*w*/*v*) agarose gels (Takara Bio Inc., Dalian, China), and then purified with a DNA gel extraction kit (BioTeke Corporation Co., Ltd, Wuxi, China) and kept at −80 °C for further operation. 

### 2.8. Pyrosequencing Data Availability for the Microbial Community Profiles

The purified PCR products from different samples were mixed in equimolar ratios based on their concentration, and then subjected to emulsion PCR for generating amplicon libraries through sequencing on a 454 Life Sciences Genome Sequencer FLX Titanium platform in the TEDA Institute of Biological Sciences and Biotechnology, Nankai University, Tianjin, China. The 454 standard flowgram format (SFF) files of the raw sequences obtained from the 454 sequencer were converted to FASTA and QUAL files using Mothur 1.10.2 [56]. Then, the raw sequence data were processed to obtain the optimized data with QIIME2-2018.11 [57], in which sequences with the following characteristics were discarded: the length exceeded bounds of 200 and 1000, number ambiguous bases exceeded 6, mean quality scores were lower than 25, maximum homopolymer run exceeded the limit of 6, number mismatches in primer exceeded 0, and/or uncorrected barcodes. All of the optimized sequences were normalized using cumulative sum scaling (CSS) [58], and then aligned to sequences on the SILVA database by BLASTN. The aligned sequences were clustered into OTUs with the furthest neighbor Jukes-Cantor distance of 0.03 (OTU_0.03_) and assigned to a taxonomy using the Ribosomal Database Project (RDP) Classifier according to Wang et al. [59]. 

### 2.9. Statistical Analysis

Statistical analyses were performed with the assistance of R (version 3.6.2; The R Foundation for Statistical Computing, Vienna, Austria). The amounts of residual oil, biomass cell densities, the pH values, and surface tension coefficients among different samples were analyzed by using the normality test with the function of KS test and the Student’s *t*-test with the function of *t*-test in stats package of R. A probability of *p* < 0.05 was considered to be significant for all tests.

## 3. Results

### 3.1. Physicochemical Characteristics Change and Microbial Community Succession after Heating Perturbation on Crude Oil at Different Frequencies

**Figure 1:**
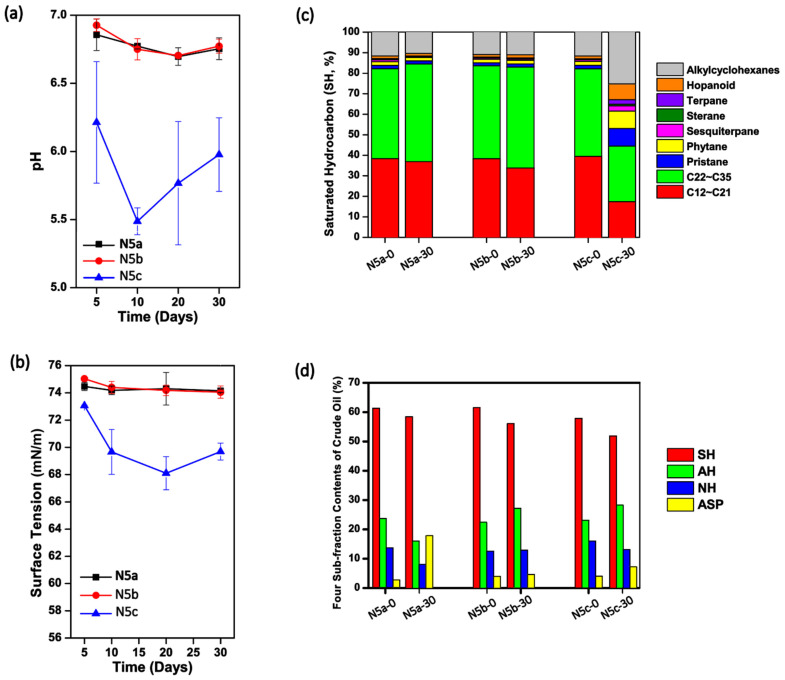
The physicochemical characteristics changes of solutions containing crude oil being pretreated with different frequencies of heating. The time courses of pH values (**a**) and surface tension degrees (**b**) in solutions containing one-time heated (N5a), two-time heated (N5b), and raw (N5c) crude oil; Comparison of the saturated hydrocarbon components (**c**) and the four-subfraction contents (**d**) in the crude oil samples collected from Treatments N5a, N5b, and N5c at the beginning and end of the 30-day incubation. SH, saturated hydrocarbon; AH, aromatic hydrocarbon; NH, non-hydrocarbons; ASP, asphaltenes.

In the study, crude oil samples collected from the Daqing Oilfield were heated once or twice at 121 °C for 20 min. Afterwards, the thermally pretreated oil samples, as well as the raw crude oil, were kept in sterilized I-SMS media at 37 °C and 150 rpm for 30 days. As shown in Figure 1a, the pH values of cultures containing once- and twice-heated crude oil, termed as N5a and N5b, respectively, were stable at 6.63–6.93 during the 30-day incubation. However, pH values in N5c (containing raw oil) were significantly lower than those in N5a and N5b (*p* < 0.05, respectively), with a fluctuation at 5.30–6.25. Similarly, the surface tension degrees in cultures of Treatments N5a and N5b were steadily high over the 30-day culture period (~74.0 mN/m), while the surface tension degrees in N5c decreased to 69.8 ± 1.8 mN/m in the first 10 days and kept at the relatively low level in the next 20 days (Figure 1b). These results indicated that the raw crude oil contained microorganisms with the function to produce acids and biosurfactants. As shown in Figure 1c, the fractions of middle-to-long chain length alkanes in raw crude oil decreased significantly in the past 30 days in Treatment N5c, suggesting that the microbes in raw crude oil preferred to utilize middle-to-long chain alkanes. By using the conventional microbe isolating methods, several microbes in the genera of *Pseudomonas*, *Microbacterium*, *Hyphomonas*, *Ochrobactrum*, *Achromobacter*, and *Rhizobium* were screened out from the solution samples in Treatment N5c. It was found that the amounts of these culture-dependent bacteria dramatically increased in the first 20 days and then slightly decreased in the next 10 days (Table S1).

Considering that the pH values (Figure 1a), surface tension degrees (Figure 1b), and saturated hydrocarbon compositions (Figure 1c) over the 30-day incubation were not significantly changed in solutions containing either once or twice thermally pretreated crude oil (*p* > 0.05), it was inferred that the microbial communities in crude oil from the Daqing Oilfield were severely perturbated during the thermal pretreatment. As shown in Table S1, neither solution samples in Treatment N5a nor those in N5b contained any strain that could grow on LB solid media, indicating that the one-time thermal pretreatment was strong enough to kill all culture-dependent microbes in raw crude oil. Nevertheless, it was believed that there should be surviving microbes with the ability to utilize different types of hydrocarbons in the thermally pretreated crude oil, especially for that in Treatment N5a, because the four sub-fraction contents of crude oil in Treatment N5a were obviously different between Day 0 and Day 30, with the relative contents of saturated hydrocarbon (SH), aromatic hydrocarbon (AH), and non-hydrocarbons (NH) decreasing while the relative content of asphaltenes (ASP) rose significantly (*p* < 0.05) (Figure 1d).

### 3.2. The Effects of Different Exogenous Bacteria on Physiochemical Characteristics Changes in the Thermally Pretreated Crude Oil

Here, two exogenous bacterial strains isolated from the Daqing Oilfield, *Amycolicicoccus subflavus* DQS3-9A1^T^ and *Dietzia* sp. DQ12-45-1b, were inoculated into sterilized I-SMS media containing either one-time heated crude oil, two-time heated crude oil with a three-day interval at 37 °C and 150 rpm, or raw crude oil. Afterwards, the cultures were shaken at 37 °C and 150 rpm for 30 days to see the effects of different exogenous bacteria on physicochemical characteristics and endogenous microbial communities in crude oil. The treatments with the inoculum *A. subflavus* DQS3-9A1^T^ were termed as A5a, A5b, and A5c, respectively, while the treatments with the inoculum *Dietzia* sp. DQ12-45-1b were termed as B5a, B5b and B5c, respectively.

**Figure 2:**
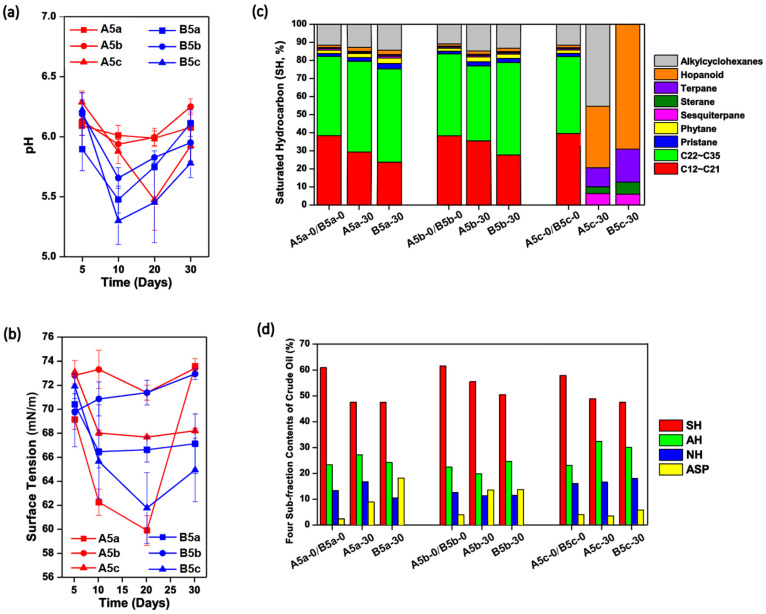
The effects of the exogenous bacterial strains *Amycolicicoccus subflavus* DQS3-9A1^T^ and *Dietzia* sp. DQ12-45-1b on physiochemical characteristics changes in cultures containing the thermally pretreated crude oil. The time courses of pH values (**a**) and surface tension degrees (**b**) in *A. subflavus* DQS3-9A1^T^ inoculated solutions containing one-time heated (A5a), twice heated (A5b), and raw (A5c) crude oil, and in *Dietzia* sp. DQ12-45-1b inoculated solutions containing one-time heated (B5a), double heated (B5b), and raw (B5c) crude oil; Histograms showing the saturated hydrocarbon components (**c**) and the four-subfraction contents (**d**) in the crude oil samples collected from Treatments A5a, A5b, A5c, B5a, B5b, and B5c at the beginning and end of the 30-day incubation. SH, saturated hydrocarbon; AH, aromatic hydrocarbon; NH, non-hydrocarbons; ASP, asphaltenes.

The physiochemical properties of each culture were monitored over the 30-day incubation. As shown in Figure 2a, the pH values in each solution decreased to 5.2–6.0 in the first 10–20 days and then rose slightly, no matter whether *A. subflavus* DQS3-9A1^T^ or *Dietzia* sp. DQ12-45-1b was injected. This trend was similar with that in Treatment N5c (Figure 1a), indicating that crude oil was partially converted to acidic compounds by microbes in these treatments. Compared with cultures inoculated with *A. subflavus* DQS3-9A1^T^, the pH decrease in cultures inoculated with *Dietzia* sp. DQ12-45-1b was more obvious. Among the six treatments, B5c reached the lowest pH level, 5.28 ± 0.22 on Day 10. These results suggested that the pH decreasing was induced by not only the exogenous bacteria but also the endogenous microbes, and *Dietzia* sp. DQ12-45-1b should be more effective than *A. subflavus* DQS3-9A1^T^ for producing acidic compounds or recovering endogenous microbes with the functions of acidic compound production. The time courses of surface tension in each treatment could explain their crude oil emulsification phenomenon over the experimental period (Figure S1). As shown in Figure 2b, the surface tension degrees for all treatments dropped dramatically in the first 20 days, except for the ones containing the two-time heated crude oil. This indicated that endogenous microbes might play important roles in biosurfactant synthesis here, but they were severely killed during the two-time thermal disturbance. Among the six treatments, A5a reached the lowest surface tension degree, 60.02±1.84 mN/m on Day 20, which was significantly lower than those in N5a (Figure 1b) and B5a (*p* < 0.05), suggesting that *A. subflavus* DQS3-9A1^T^ could produce biosurfactants and its productivity was higher than *Dietzia* sp. DQ12-45-1b. Considering that the surface tension degrees of A5a were significantly lower than those in A5c (*p* < 0.05), it was inferred that *A. subflavus* DQS3-9A1^T^ should have amensalistic interactions with some endogenous microbes and commensalistic interactions with some other ones in raw crude oil, and after the one-time thermal pretreatment, the amounts of survival endogenous microbes which had negative effects on *A. subflavus* DQS3-9A1^T^ were less than those with positive effects. Among the four culture systems with thermal pretreatments, only B5a had a continuous decrease of the surface tension degrees toward stability, and its final surface tension degree was significantly lower than those in A5a, A5b, and B5b, respectively (*p* < 0.05), suggesting that *Dietzia* sp. DQ12-45-1b was sustainably effective at activating biosurfactant-producing endogenous microbial survivors from one-time heated crude oil.

For the SH components, it was found that only residual oil in A5c and B5c had obviously different SH compositions to the initial crude oil. As shown in Figure 2c, medium-, long-, and extra-long-chain alkanes, phytane and pristane, were removed completely in both A5c and B5c, and alkylcyclohexanes were also consumed completely in B5c. These results indicated that the significant changes of the SH compositions were caused by SH-degrading endogenous microbes in raw crude oil rather than the exogenous microbes, and the thermal pretreatment on crude oil prevented SH biodegradation. Nevertheless, the relative contents of SH in crude oil decreased over the 30 days, regardless of crude oil thermal pretreatment severity and exogenous bacterial species (Figure 2d). Interestingly, the relative decline of SH fractions was obviously less in Treatment A5b in comparison with those in other treatments, suggesting that *A. subflavus* DQS3-9A1^T^ and the very few endogenous microbes in the two-time thermally pretreated crude oil, if present, were passive for SH utilization.

### 3.3. The Effects of Different Exogenous Bacteria on the Microbial Community Reconstruction in the Thermally Pretreated Crude Oil

The microbial community compositions in treatments A5a, A5b, A5c, B5a, B5b, and B5c over time were determined in the following three ways: (1) accounting for the culture-dependent bacteria using CFU testing, (2) identifying the bacterial community structures using 16S rRNA gene amplicon sequencing, and (3) determining the active community structures using sequencing of 16S rRNA gene amplicon generated by reverse transcription-PCR.

**Figure 3:**
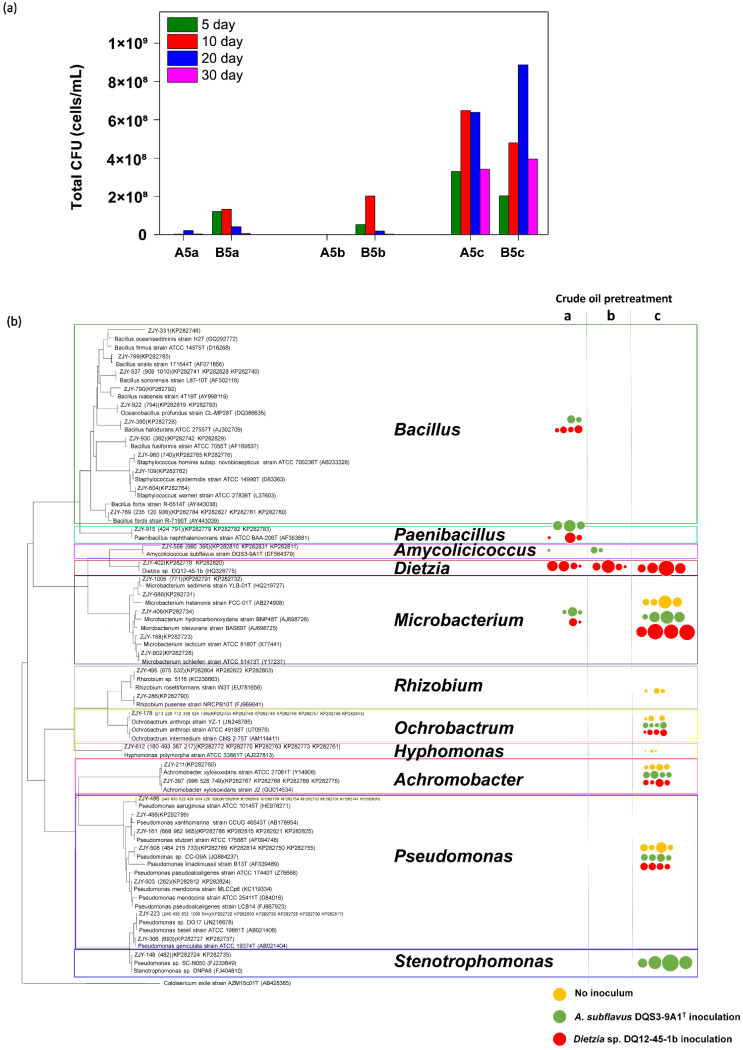
Effects of different exogenous bacteria on culture-dependent microbial community reconstruction in thermally pretreated crude oil based on the CFU-testing data. (**a**) The histograms showing cell densities of total culture-dependent bacterial cells over the 30-day incubation in *A. subflavus* DQS3-9A1^T^ inoculated culture systems containing one-time heated (A5a), double heated (A5b), and raw (A5c) crude oil, and in *Dietzia* sp. DQ12-45-1b inoculated solutions containing one-time heated (B5a), twice heated (B5b), and raw (B5c) crude oil. (**b**) The phylogenetic tree of representative bacterial colonies isolated from samples of each culture system on Day 5, Day 10, Day 20, and Day 30, with their cell densities being represented in small to big circles. Orange circles, treatments without inoculum; green circles, culture systems inoculated by *A. subflavus* DQS3-9A1^T^; red circles, culture systems inoculated by *Dietzia* sp. DQ12-45-1b; thermal pretreatment manner ‘a’, one-time heating; manner ‘b’, two-time heating with a three-day interval at 37 °C; manner ‘c’, no heating.

Inevitably, the cell densities of culture-dependent bacteria in culture systems containing thermally pretreated crude oil were orders of magnitude lower than those in A5c and B5c (Figure 3a). It was noticed that the culture-dependent bacterial biomass was more successfully stimulated by *Dietzia* sp. DQ12-45-1b than that by *A. subflavus* DQS3-9A1^T^, regardless of thermal pretreatment with different frequencies on crude oil or not. To see what types of culture-dependent microbes were present, the taxonomies of representative colonies were addressed in each treatment and a phylogenetic tree with the responding cell density data was developed. As shown in Figure 3b, the raw crude oil contained culture-dependent bacterial isolates in genera *Microbacterium*, *Rhizobium*, *Ochrobactrum*, *Hyphomonas*, *Achromobacter* and *Pseudomonas*, and their amounts had a trend of increasing first and then decreasing during the 30-day incubation at 37 °C. Nevertheless, *Rhizobium* strains and *Hypomonas polymorpha* might have amensalistic interactions with the exogenous bacteria, because they were no longer detected in treatments A5c and B5c. The heat shock killed most of the isolates in situ, excepting *Microbacterium* species. However, it was surprisingly found that some genera strains which were not detected from raw crude oil were stimulated by the exogenous bacteria on the one-time heated crude oil, such as *Bacillus* strains and *Paenibacillus naphthalenovorans* strains, suggesting that these strains had a high temperature tolerance but were minor or dormant in raw crude oil. Meanwhile, it was found that endogenous bacterial strains being able to proliferate on double heated crude oil were very few, no matter whether *A. subflavus* DQS3-9A1^T^ or *Dietzia* sp. DQ12-45-1b was injected. 

**Figure 4:**
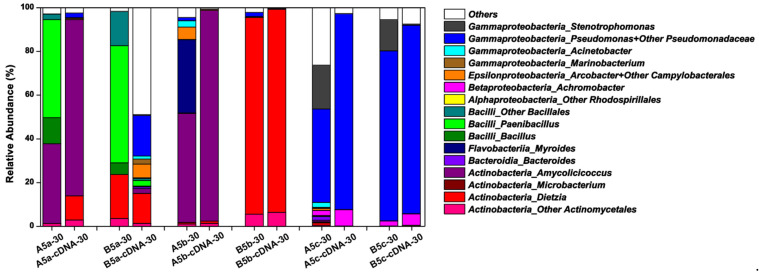
The histograms showing the total microbial community structures in treatments A5a, B5a, A5b, B5b, A5c, and B5c after the 30-day incubation. Treatment No.X-30, the bacterial community structures of Treatment No.X on Day 30 based on 16S rRNA gene amplicon sequencing; Treatment No.X-cDNA-30, the active community structures of Treatment No.X on Day 30 based on sequencing of 16S rRNA gene amplicon generated by reverse transcription-PCR.

Considering that most microbes in natural microbial communities were culture-independent, 16S rRNA gene amplicon sequencing were operated to learn about the whole microbial community compositions in each harvested culture solution. Partially in accordance with the CFU testing-based taxonomy data, the microbial community structures were similar in A5c and B5c after the 30-day incubation, in which *Pseudomonas* followed by *Stenotrophomonas* and *Achromobacter* were the predominant genera, regardless of sequencing data from DNA or cDNA samples (Figure 4). *Pseudomonas*, *Stenotrophomonas* and *Achromobacter* became minor genera in microbial communities constructed on thermally pretreated crude oil. For treatments containing the double heated crude oil, the active microbial communities on Day 30 were mainly composed of the exogenous genera, including *Amycolicicoccus* for A5b and *Dietzia* for B5b. Compared with *Dietzia* sp. DQ12-45-1b, *A. subflavus* DQS3-9A1^T^ could stimulate more types of endogenous microbes in crude oil after the double heating process, but they were mainly at the dormant status, such as *Myroides*, *Acrobacter*, other *Campylobacterales*, *Acinetobacter*, and *Pseudomonas*. These results confirmed that the double heating process was strong enough to kill most microbes that existed in crude oil. For treatments containing the one-time heated crude oil, the microbial communities were mainly composed of *Paenibacillus*, *Bacillus*, other *Bacillales*, and the exogenous genera, which was essentially consistent with the CFU testing-based taxonomy data (Figure 3b). However, *Paenibacillus*, *Bacillus*, and other *Bacillales* became minor ones in A5a and B5a cDNA samples, indicating that these genera in the constructed bacterial community were at the dormant status. Compared with other treatments, B5a had the most complicated active microbial community, in which unidentified genera occupied around 50% of the whole active community and more than 10 identified endogenous genera beside with the exogenous genus *Dietzia* were enriched. Therefore, it was indicated that *Dietzia* sp. DQ12-45-1b was very effective at inducing the proliferation of varied species in one-time heated crude oil, and could be potentially utilized for MEOR in thermal recovery-processed oil reservoirs.

### 3.4. Effects of Salinity on Detzia sp. DQ12-45-1b Induced Bioemusification and Microbial Community Reconstruction in Heated Crude Oil

The salinity in oil reservoirs was a second important environmental factor for microbial growth in situ. Here, to see if crude oil bioemulsification through MEOR could still effectively occur under the stress of reparable heat perturbation and high salinity, *Dietzia* sp. DQ12-45-1b was inoculated into the hypersaline I-SMS medium (50 g/L NaCl) containing either thermally pretreated crude oil or raw crude oil for a 30-day incubation, and these treatments were termed as B50a and B50c, respectively.

**Figure 5:**
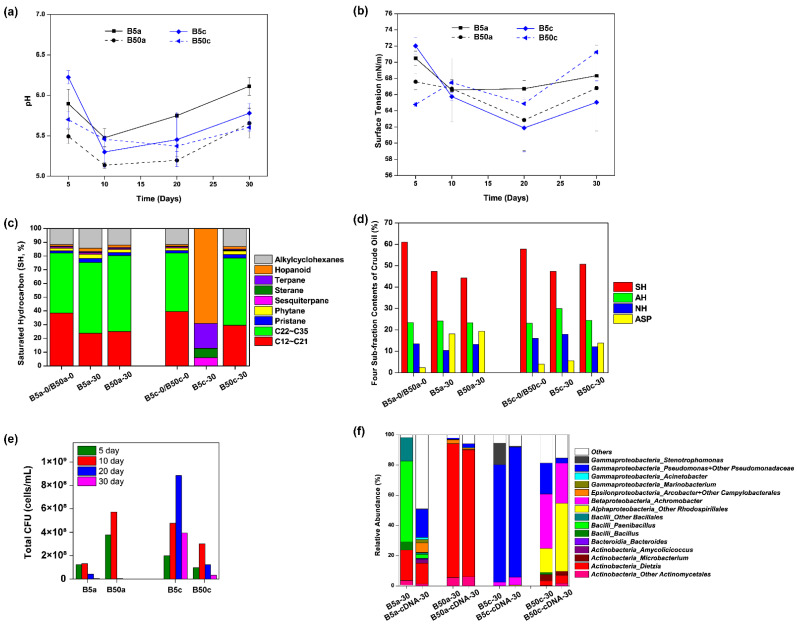
Effects of high salinity on *Dietzia* sp. DQ12-45-1b-induced physiochemical characteristics changes and microbial community reconstruction in crude oil. Time courses of pH values (**a**) and surface tension degrees (**b**) in *Dietzia* sp. DQ12-45-1b inoculated normal and hypersaline solutions containing either one-time heated (B5a and B50a, respectively) or raw (B5c and B50c, respectively) crude oil. Histograms showing the saturated hydrocarbon components (**c**) and the four-subfraction contents (**d**) in the crude oil samples collected from treatments B5a, B50a, B5c, and B50c at the beginning and end of the 30-day incubation; SH, saturated hydrocarbon; AH, aromatic hydrocarbon; NH, non-hydrocarbons; ASP, asphaltenes. Histograms showing cell densities of total culture-dependent bacterial cells over the 30-day incubation (**e**) and total microbial community structures based on DNA or cDNA sequencing at the end of the incubation (**f**) in treatments B5a, B50a, B5c, and B50c.

As shown in Figure 5a, pH values in B50a and B50c were relatively stable and significantly lower than those in the normal salinity (5 g/L NaCl) over the time, ranging at 5.14–5.66 for B50a and 5.37–5.70 for B50c. Each culture system had photographs taken on Day 5, Day 10, Day 20, and Day 30, and it was observed that crude oil in B50a and B50c became well dispersive on Day 5 (Figure S1). As shown in Figure 5b, the surface tension degrees in B50a and B50c had the same trend of first decreasing and then increasing, and their values were not significantly different from each other (*p* > 0.05) or from those in B5a and B5c, respectively (*p* > 0.05), indicating that *Dietzia* sp. DQ12-45-1b might be still effective for crude oil bioemulsification when the cells are in reparable heat perturbated microbial communities in situ and/or high salinity. After the 30-day incubation, although the SH composition of residual crude oil had no significant change in B50a and B50c (Figure 5c), the relative contents of SH and ASP altered dramatically in B50a and B50c, with the SH fraction decreasing and the ASP fraction increasing, especially for the residual oil in B50a (Figure 5d). Since residual oil samples in B50a and B5a had similar SH composition profiles as did the four sub-fraction contents at the end of the incubation, it was inferred that the hypersalinity stress had few effects on *Dietzia* sp. DQ12-45-1b induced microbial community reconstruction in one-time heated crude oil. Nevertheless, as shown in Figure 5e, the culture-dependent bacterial biomass in B50a were much more accumulated than those in B5a in the first 10 days. According to the following representative colony taxonomy identification, the types of bacteria in B50a were very few, including few species in genera *Bacillus*, *Microbacterium*, and *Dietzia*. The colony amounts of *Dietzia* strains were predominant in B50a and were obviously more than those in B5a in the first 10 days. In accordance with the CFU-testing results, 16S rRNA gene amplicon sequencing analysis showed that the microbial community in B50a on Day 30 was very different from those in B5a and B50c, and was mainly composed of *Dietzia*, no matter whether the sequencing was based on DNA samples or cDNA samples (Figure 5f). Even though crude oil bioemulsification and acidification effects in B50a were similar with those in B5a and B50c, their internal metabolic mechanisms were varied. All above results indicated that crude oil bioemulsification and acidification in B50a were mainly due to the metabolic functions of *Dietzia* sp. DQ12-45-1b. 

## 4. Discussion

Petroleum reservoirs are harsh habitats for microorganisms because of the high toxicity and hydrophobicity. However, multiple taxonomic microorganisms adapted well in oil reservoirs and have been identified in situ [60,61,62,63]. Meckenstock et al. [64] proved that microorganisms can live in minuscule water droplets entrapped in crude oil. However, since large amounts of new electron donors, acceptors, and/or exogenous microbes are injected into the subsurface biosphere through different oil exploration processes, the relatively stable microbiomes in the deep reservoir environment are often reshaped. For example, Vigneron et al. [65] investigated the microbial community succession in 32 producing wells of Halfdan oil field, and found that after the 15-year oil exploration using seawater and nitrate injection, the predominant microbes altered from slow-growing anaerobes, such as *Thermotogales* and *Clostridiales*, to fast-growing opportunists which preferred the energetically more favorable metabolisms of nitrate reduction or sulfide oxidation, such as *Deferribacteres*, *Delta-*, *Epsilon-* and *Gammaproteobacteria*. In our study, it was found that the thermal process on crude oil could severely perturbate endogenous microbiome, destroying most culture-dependent species in situ (Figure 3b), but the negative effects of heating perturbation was made up by the locally isolated bacterial strain *Dietzia* sp. DQ12-45-1b. 

As shown in Figure 3b and Figure 4, thanks to the addition of *Dietzia* sp. DQ12-45-1b, some endogenous microbial survivors from heating, such as the spore-forming genera of *Bacillus* and *Paenibacillus* and non-spore-forming *Pseudomonas*, became activated and reorganized to form a complicated bacteria community. Meanwhile, the surface tension degrees in the *Dietzia* sp. DQ12-45-1b-inoculated culture system had a relatively stable decrease during the incubation, and reached the lowest point among the treatments containing thermally pretreated crude oil (Figure 2b). All above results suggested that *Dietzia* sp. DQ12-45-1b could be potentially utilized for MEOR in thermal recovery-processed oil reservoirs. Further, it has already been realized that, compared with other microorganisms, locally isolated microbes can adapt to extreme environmental reservoirs better, and thus are considered to be good candidates for MEOR [66,67,68]. For example, Rathi et al. [37] enriched a methanogenic consortium TERIL146 consisting of *Methanothermobacter* sp., *Thermoanaerobacter* sp., *Gelria* sp. and *Thermotoga* sp. from a 70 °C oil reservoir in India, and found that it had 8.3% incremental oil recovery in thermophilic sandpack assay, indicating that TERIL146 could be utilized for oil recovery in thermophilic depleted wells. Nevertheless, we are the first ones to investigate if the MEOR candidates isolated from a mesophilic oil field could be utilized for the bioemulsification and biodegradation of thermally processed crude oil collected from the same oil field. Our study provides useful information to understand MEOR trials on thermally processed oil reservoirs, and proves that this strategy could be operated by using the locally available hydrocarbon-degrading and biosurfactant-producing microbes at mesophilic conditions. 

Temperature seems to be the most important factor for MEOR efficiency [33], followed by salinity [69]. Larter et al. [70] state that salinity had a second-order effect slowing hydrocarbon biodegradation. However, as shown in Figure 5a–d, the hypersalinity (50 g/L NaCl) had few negative effects on crude oil bioemulsification, acidification, and residual oil composition alteration in the study. Considering that the microbial community compositions in B50a and B5a were obviously different (Figure 5e,f), it was inferred that *Dietzia* sp. DQ12-45-1b was tolerant to hypersalinity under the existence of thermally pretreated crude oil, but the endogenous microbial survivors with amensalistic interactions with *Dietzia* sp. DQ12-45-1b were severely inhibited under the hypersaline conditions. Fang et al. [71] found that *Dietzia* sp. DQ12-45-1b had hypersaline and alkaline resistance due to the existence of the six-subunit Na^+^/H^+^ antiporter DqMrp in its genome.

For ecosystems consisting of plants and/or animals, species are classified into two types based on species life-histories: r- and K-strategists [72]. For r-strategists, they allocate more energy to reproduction instead of biomass accumulation and competition, so species with r-selection strategy can rapidly establish on free sites with great colonizing ability when resources are abundant. In contrast, K-strategists produce high biomass with strong competitive ability when the carrying capacity of populations and resources are limited [73,74]. In microecosystems, ‘copiotroph’ and ‘oligotroph’ were defined to describe microorganisms with ecological attributes typical of r- and K-strategists, respectively [75]. In our study, r-strategists preferentially survive with adequate crude oil and fewer toxic metabolites, have high nutritional requirements, and can exhibit high growth rates. When endogenous microorganisms are r-strategists, they will easily flourish, regardless of exogenous microorganisms, and inhibit other endogenous microorganisms such as *Pseudomonas* in N5c, A5c, and B5c. In contrast, K-strategists exhibit slower growth rates and are likely to outcompete copiotrophs in conditions of limited crude oil and more survival stresses, such as *Achromobacter* and *Microbacterium* in B50c. 

In general, some endogenous microbes in crude oil could survive one-time heat perturbation, and proliferate or become stronger due to the introduction of locally isolated bacterial strain *Dietzia* sp. DQ12-45-1b with the capabilities of biosurfactant production and hydrocarbon degradation under mesophilic conditions, and then interact with *Dietzia* sp. DQ12-45-1b to achieve enhanced crude oil emulsification. Even though the characteristics and functions of microorganisms in thermally pretreated crude oil have been gradually understood, the relationships among microorganisms for the guidance on MEOR and bioremediation are still unclear and are required to be explored in the future.

## 5. Conclusions

In this study, microhabitats in crude oil collected from the No. 3 oil product of the Daqing Oilfield contained diverse microorganisms in situ, such as *Pseudomonas*, *Microbacterium*, *Hyphomonas*, *Ochrobactrum*, *Achromobacter*, and *Rhizobium*. The microbial community perturbation caused by the thermal treatment was partially recovered by either *A. subflavus* DQS3-9A1^T^ or *Dietzia* sp. DQ12-45-1b. Compared with *A. subflavus* DQS3-9A1^T^, *Dietzia* sp. DQ12-45-1b was more effective at inducing culture acidification, crude oil bioemulsification, and the proliferation of varied species in one-time heated crude oil during the 30-day incubation. Meanwhile, it was found that the hypersalinity stress (50 g/L NaCl) had few negative effects on *Dietizia* sp. DQ12-45-1b induced crude oil bioemulsification, acidification, and residual oil composition alteration. All above results indicated that the locally isolated bacterial strain *Dietzia* sp. DQ12-45-1b, which had strong hydrocarbon-degrading capability and hypersaline resistance, could be potentially utilized for MEOR in thermal recovery-processed oil reservoirs with mesophilic conditions, regardless of high or low salinity degrees.

In order to make the thermal-microbial EOR methods more applicable soon, further studies of the hybrid processes should aim to characterize and clarify the metabolic interactions among the endogenous and exogenous microbes during the hybrid recovery processes.

## Figures and Tables

**Table 1:** Summary of the experimental scheme.

Treatment Label	Culture Thermal Pretreatment	NaCl in I-MSM (g/L)	Exogenous Bacteria
N5a	Autoclaving at 121 °C for 20 min	5	-
N5b	Twice autoclaving at 121 °C for 20 min with a three-day interval	5	-
N5c	-	5	-
A5a	Autoclaving at 121 °C for 20 min	5	*A. subflavus* DQS3-9A1^T^
A5b	Twice autoclaving at 121 °C for 20 min with a three-day interval	5	*A. subflavus* DQS3-9A1^T^
A5c	-	5	*A. subflavus* DQS3-9A1^T^
B5a	Autoclaving at 121 °C for 20 min	5	*Dietzia* sp. DQ12-45-1b
B5b	Twice autoclaving at 121 °C for 20 min with a three-day interval	5	*Dietzia* sp. DQ12-45-1b
B5c	-	5	*Dietzia* sp. DQ12-45-1b
B50a	Autoclaving at 121 °C for 20 min	50	*Dietzia* sp. DQ12-45-1b
B50b	Twice autoclaving at 121 °C for 20 min with a three-day interval	50	*Dietzia* sp. DQ12-45-1b
B50c	-	50	*Dietzia* sp. DQ12-45-1b

## Data Availability

The pyrosequencing data presented in the study has been deposited to DNA Data Bank of Japan (DDBJ; https://www.ddbj.nig.ac.jp/index-e.html), and is openly available under the accession numbers of DRA002851 in DDBJ.

## Supplementary Materials

The following are available online at https://www.mdpi.com/article/10.3390/microorganisms9102054/s1, Table S1: Cell densities of total culture-dependent bacterial cells in each treatment over the 30-day incubation determined by the CFU testing, Figure S1: Photographs showing the culture status in each treatment over the 30-day incubation.
