# Supplementary material for: Bioemulsification and Microbial Community Reconstruction in Thermally Processed Crude Oil
Source: Microorganisms. 2021 Sep 29;9(10):2054. doi: 10.3390/microorganisms9102054 (PMC8539444; doi:10.3390/microorganisms9102054)
Supplement: Supplementary file 1 [file microorganisms-09-02054-s001.zip › Figure S1.pdf]

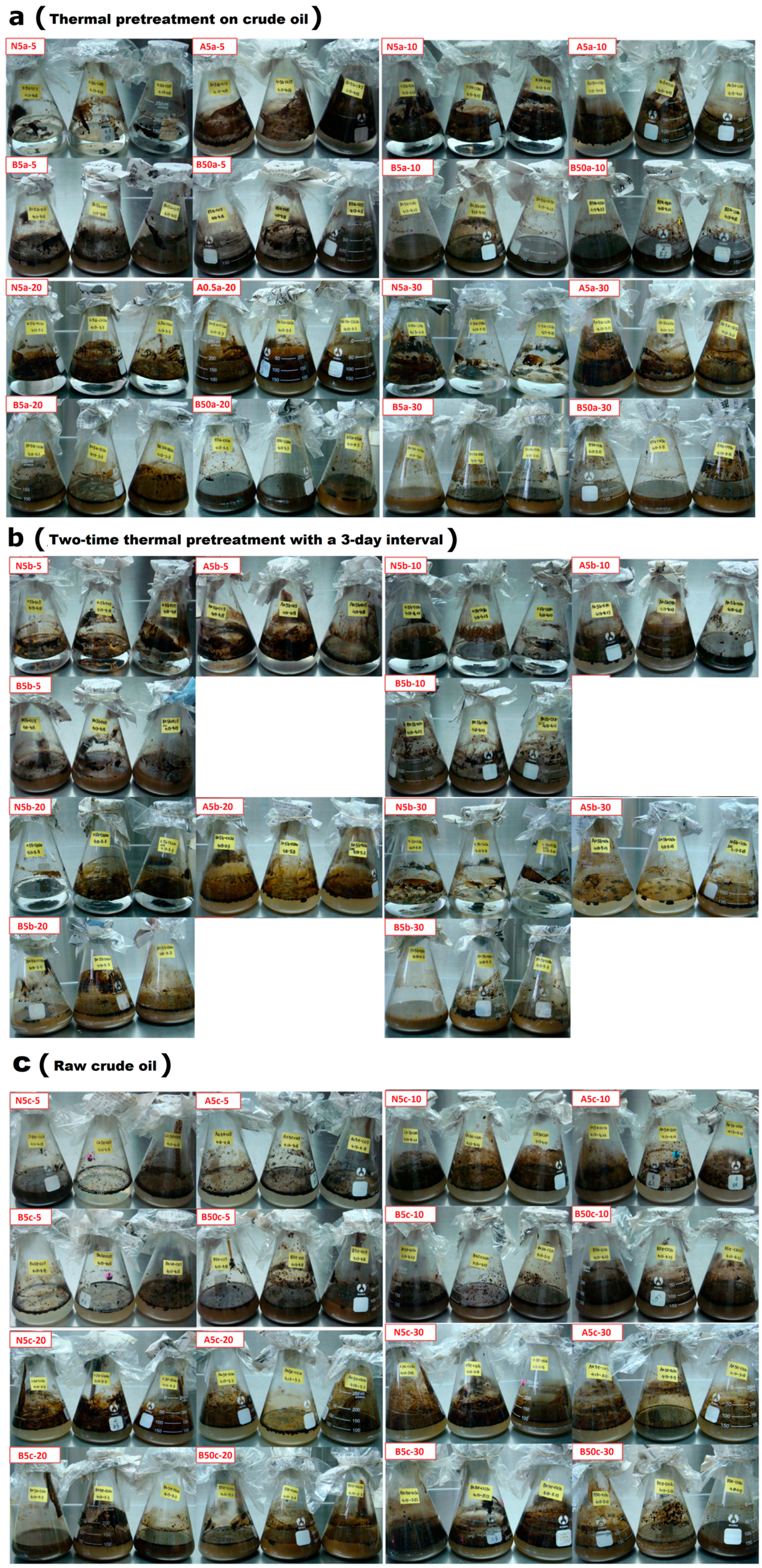

Figure S1: Photographs showing the culture status in each treatment over the 30-day incubation. (**a**) Thermal pretreatment on crude oil. (**b**) Two-time thermal pretreatment with a 3-day interval. (**c**) Raw crude oil.
